# A method for transforming knowledge discovery metamodel to ArchiMate models

**DOI:** 10.1007/s10270-021-00912-y

**Published:** 2021-08-02

**Authors:** Ricardo Pérez-Castillo, Andrea Delgado, Francisco Ruiz, Virginia Bacigalupe, Mario Piattini

**Affiliations:** 1 Faculty of Social Sciences and IT, University of Castilla-La Mancha, Av. Real Fábrica de Sedas s/n, 45600 Talavera de La Reina, Spain; 2 Instituto de Computación, Facultad de Ingeniería, Universidad de La República, 11300 Montevideo, Uruguay; 3 Information Technology and Systems Institute (ITSI), University of Castilla-La Mancha, Paseo de la Universidad 4, 13071 Ciudad Real, Spain

**Keywords:** Enterprise architecture, ArchiMate, Knowledge discovery metamodel, Model transformation, MDE, ATL

## Abstract

Enterprise architecture has become an important driver to facilitate digital transformation in companies, since it allows to manage IT and business in a holistic and integrated manner by establishing connections among technology concerns and strategical/motivational ones. Enterprise architecture modelling is critical to accurately represent business and their IT assets in combination. This modelling is important when companies start to manage their enterprise architecture, but also when it is remodelled so that the enterprise architecture is realigned in a changing world. Enterprise architecture is commonly modelled by few experts in a manual way, which is error-prone and time-consuming and makes continuous realignment difficult. In contrast, other enterprise architecture modelling proposal automatically analyses some artefacts like source code, databases, services, etc. Previous automated modelling proposals focus on the analysis of individual artefacts with isolated transformations toward ArchiMate or other enterprise architecture notations and/or frameworks. We propose the usage of Knowledge Discovery Metamodel (KDM) to represent all the intermediate information retrieved from information systems’ artefacts, which is then transformed into ArchiMate models. Thus, the core contribution of this paper is the model transformation between KDM and ArchiMate metamodels. The main implication of this proposal is that ArchiMate models are automatically generated from a common knowledge repository. Thereby, the relationships between different-nature artefacts can be exploited to get more complete and accurate enterprise architecture representations.

## Introduction

Enterprise architecture (EA) is a key mechanism to represent and manage IT and business in a holistic way by defining relationships between technology aspects and business, strategical, and motivational concerns. EA management (EAM) is the “discipline for proactively and holistically leading enterprise responses to disruptive forces by identifying and analysing the execution of change toward desired business vision and outcomes. EA delivers value by presenting business and IT leaders with signature-ready recommendations for adjusting policies and projects to achieve target business outcomes that capitalize on relevant business disruptions” [16]. One of the major benefits of EAM perceived by companies is that it enables them to achieve the effective communication and alignment between business and IT [29], and drive the organization change [2]. Thus, EA is now perceived by companies as on the most useful tools to drive digital transformation, i.e., a technology-driven continuous change process of companies and our entire society [49].

Although the alignment of the business and IT can be achieved through EA models, such models must be revisited continuously, due to the agile adaptation of companies within changing markets and volatile technologies [67]. Companies are consequently forced to (re)define business goals and processes, along with the respective functionality of their IT stacks, by (re)developing and operating them in a continuous way [10, 12]. As a consequence, EA modelling has become in one of the most critical tasks within EAM [51]. EA modelling has traditionally been carried out manually by experts. However, manual EA modelling has several flaws [48], such as error-proneness, time-consumption, slow and poor re-adaptation, and cost. In most of the cases, the main reason for such problems lies in the subjective opinion provided by experts when they create EA models, which might lead to models with missing elements and irrelevant elements. Thereby, some researchers have claimed the need to automate EA modelling through the use of different reverse engineering and mining techniques in order to discover EA models [12, 14, 47].

**Fig. 1:**
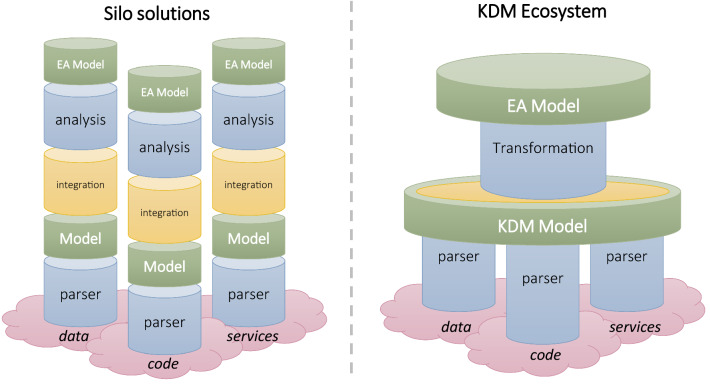
Comparison of EA mining techniques by using (or not) Knowledge Discovery Metamodel

EA elements, according to different EA viewpoints, can be extracted from a wide variety of artefacts (e.g., information systems, enterprise service bus, databases, source code, etc.). Current proposals provide techniques and tools that focus on specific artefacts and generate certain EA elements and relationships in the same or various EA models. Most of the existing techniques are thus built as a silo solution (see left hand side in Fig. 1). In bottom-up silo solutions, different parsers or alternative mining methods are used to extract information from various independent artefacts; then different platform-specific models are built for every artefact. In silo solutions, various models may be integrated for the same artefact, while these silos are independent among them. Finally, some analysis methods can be applied to synthetize some information and abstract it into the target EA models. This signifies that the specific information extracted or generated by mining techniques are used independently for different analysers and transformations to generated certain EA viewpoints in isolation. What we propose in this research is the usage of Knowledge Discovery Metamodel (KDM) [45], according to a Model-Driven Engineering (MDE) approach, in order to consider a common knowledge repository that can be used in an integrated way for automatic transformations. MDE can boost the automatic EA modelling since abstract representations of IT artefacts can be reused by automatic model transformations. In contrast, KDM ecosystems (see Fig. 1) facilitate the definition of EA model transformations based on a standard notation that allow to abstract specific reverse engineering details for all the specific artefacts. This idea is similar to the work proposed by [28] who introduced an integration layer for the automation of EA models that synchronizes static and runtime data from different data sources. The advantage of KDM is that many existing reverse engineering and mining tools use this standard and may be reused for EA modelling through the KDM to ArchiMate transformation proposed in this paper.

The main contribution of this paper is the definition of a model transformation from KDM to EA models (represented according to ArchiMate). The transformation focuses on source code of information systems that is reversed into KDM models that are then transformed into ArchiMate by considering the application and technology layers of the standard. The model transformation is implemented in ATL and evaluated in a case study with KDM models obtained from the source code of a real-life information system. The main implication of this work is that the feasibility and suitability of a model-driven engineering approach for EA modelling are demonstrated through the definition and application of automatic model transformation. As a result, software representation in ArchiMate allows to represent what that business context is, and specifically, the relationship between a technology solution and the business context. Thus, EA modelling can be boosted by automating some modelling tasks while flaws associated with manual modelling are reduced. As a result, EA models can be continuously updated in an easier way, and the alignment of business and IT is therefore improved. This eventually allows companies to make better business/IT decisions.

The remaining of the paper is structured as follows. Section 2 introduces the core concepts used in the paper. Section 3 discusses some related work. Section 4 explains in detail the KDM to ArchiMate transformation. Section 5 demonstrates the applicability of the model transformation in a proof-of-concept and with a case study involving six open-source systems. Section 6 evaluates the proposal through the analysis of results obtained in the case study. Finally, Sect. 7 draws conclusions and future work.

## State of the art

This section introduces the main concepts involved in the research proposal. First, the core concepts of model-driven engineering are summarized. Second, ArchiMate is presented as the de facto standard for representing and managing EA models, which is used as the output metamodel. Last, the KDM standard is presented, which is used as the input metamodel in the proposed transformation.

### Model-driven engineering

MDE focuses on models as centre of the software development process, being models the most important artefacts from which other models and code are generated [27, 55, 64]. Models, metamodels and transformations between them are key elements in the process of MDE, being the basis for understanding, specifying, and analysing software systems. Metamodels define modelling languages (abstract syntax) providing concepts and relationships between them, and notations (concrete syntax) that can be graphical or textual, in order to specify models that represent those systems [26, 52]. Examples of such modelling languages are the Unified Modelling Language (UML) [38], Business Process Model and Notation (BPMN 2.0) [34], ArchiMate, and KDM. Meta-metamodels allow the definition of modelling languages to specify metamodels, such as the Metamodel Object Facility (MOF) [37] and *Ecore*, its technological implementation on Eclipse platform. Models specified in a modelling language “conform to” the corresponding metamodel, i.e., all concepts and relationships specified in the model are as defined by the metamodel.

**Fig. 2:**
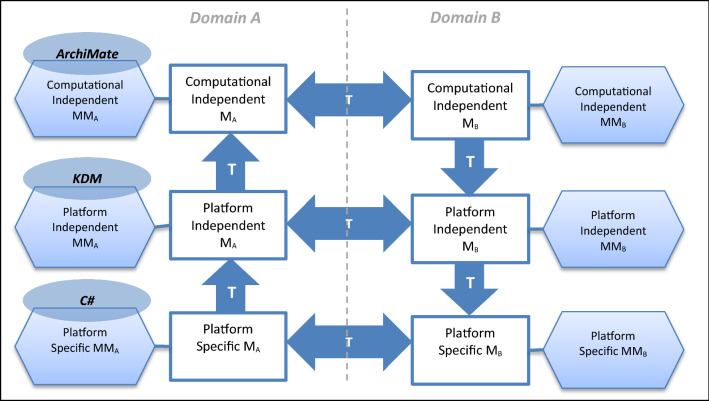
Possible transformations in MDA context

MDE can provide, based on model transformations, refinement steps that decrease the level of abstraction usually traveling from specification models to code, but also allowing other scenarios such as reverse engineering, i.e., traveling from code to models [26], helping to recover the hidden knowledge (see Fig. 2). Other model transformations consider models at the same abstraction levels but in different domains (see Fig. 2). The model-driven architecture (MDA) [35] is a specific implementation of MDE provided by the Object Management Group (OMG) to support the development of systems based on transformation of models from specifications to code. In this context, the architecture-driven modernization (ADM) [39] approach supports the reengineering of information systems going from code to specification models.

To specify transformations, specific-purpose languages are also needed. Examples are Query/Views/Transformations (QVT) [33] which defines two declarative languages (QVT Core and QVT Relations) and an imperative language (QVT Operational),as well as ATLAS Transformation Language (ATL) [26] which provides a mixture of declarative and imperative constructs, where the declarative style allows specifying relations between source and target patterns between elements from the metamodels in the transformation, being closer to the way developers perceived it. The imperative style allows using well-known constructs for specifying control flow elements such as loops, conditions, among others.

Transformations follow a common pattern known as *model transformation pattern*, where T_AB_ is a model transformation between domains *A* and *B*. The execution of T_AB_ takes as input the model M_A_ and generates the model M_B_. M_A_, T_AB_ and M_B_ are models that, respectively, conform metamodels MM_A_, MM_T_ and MM_B_ (see Fig. 2). In turn, those three metamodels conform to the MMM meta-metamodel [26]. In the context of MDA, the MMM meta-metamodel is the Meta Object Facility (MOF). Additionally, a model transformation may occur within the same domain to reduce or increase abstraction, for example, from a Computational Independent Model CIM_B_ to a Platform Independent Model PIM_B_ (see Fig. 2).

### ArchiMate

With the increasing interest on EA, various EA frameworks have been appeared and some of them have gained certain adoption in the industry such as TOGAF, DoDAF or MODAF, Zachman, ESARC, etc. The TOGAF framework [58] is extensively adopted by private companies and can be said that it is the de facto standard [57, 60]. TOGAF proposes the Architecture Development Method (ADM) as an iterative methodology for defining EA.

**Fig. 3:**
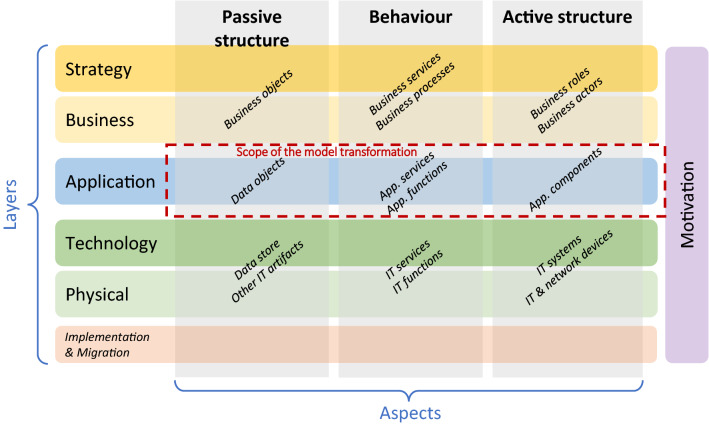
ArchiMate 3 core framework (adapted from [59])

EA modelling languages and specifications are necessary, alongside EA frameworks, to depict all the EA concerns in different architectural viewpoints. ArchiMate [59] is a modelling language compliant with TOGAF with which to represent different EA information models. ArchiMate allows the modelling of EA from different viewpoints, in which the position within the cells highlights the stakeholders’ concerns (see Fig. 3).

ArchiMate proposes layers and aspects as the two main dimensions for organizing all the elements. Core layers represent the three levels at which it is possible to model an enterprise in ArchiMate, i.e., business, application, and technology. Aspects refers to: (i) the active structure (elements representing who/what makes the things), (ii) behaviour (elements indicating what is made and how it is made), and (iii) passive structure (things on which behaviour is performed). Despite the mentioned structure, composite elements belonging to various aspects are also allowed. Finally, it should be noticed that the ArchiMate specification define further layers for strategy, physical and implementation/migration elements, as well as fourth additional aspects with motivational elements (why things are made). The scope of the model transformation of this research consists of the application layer (see highlighted part in Fig. 3).

There are several EA suites that facilitate the manual modelling of ArchiMate models [49]. In this paper, we mostly are based on Archi tool [4], an open source ArchiMate modelling tool that is based on Eclipse project and, therefore, it provides the ArchiMate *Ecore* metamodel.

### Knowledge discovery metamodel

KDM, recognized as standard ISO/IEC 19506, makes it possible to represent all software artefacts involved in a certain legacy information system in an integrated and standardized way [45]. This metamodel was specifically defined to be used within the architecture-driven modernization (ADM) approach [39], i.e., reengineering of information systems following the (MDE) principles. A KDM model is obtained in an integrated manner because it works as a KDM repository that can be gradually completed with knowledge discovered through the analysis of different information systems and different artefacts. Thus, KDM avoid silo solutions where different miners, analysers and transformations operate in isolation (see Fig. 1).

**Fig. 4:**
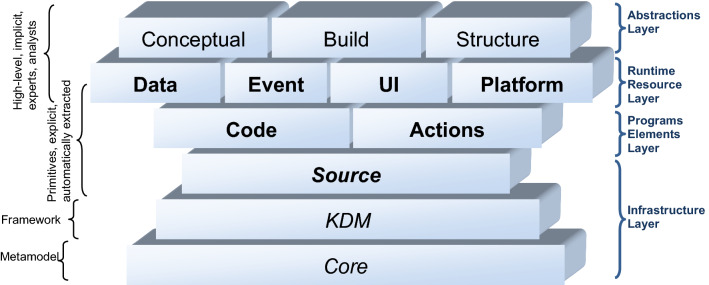
Layers, packages and concerns in KDM (Adapted from [36])

The KDM metamodel provides a comprehensive high-level view of the behaviour, structure, and data of systems, while procedural information of the systems (i.e., sequence and control flow in source code) is not the main purpose of KDM. Such kind of information is better represented by using other standards like UML. The KDM metamodel is divided into layers representing both physical and logical software artefacts of information systems at several abstraction levels [36]. It separates knowledge about legacy information systems into various orthogonal concerns that are known in software engineering as architecture views (see Fig. 4). The KDM metamodel consists of four abstraction layers, each based on a previous layer. Furthermore, each layer is organized into packages that define a set of metamodel elements whose purpose is to represent a specific independent facet of knowledge related to information systems.

## Related work

There is certain research about reverse engineering of EA models. Such works consider, as input, a wide range of information systems artefacts [47]. For example, [14, 15] automate the collection of relevant data from various external sources. It provides a specific metamodel and draws some techniques to achieve a better synchronization between EA models and organizations’ facts. Also, Kleehaus and Matthes [28] leverage runtime service instrumentation of the existing IT architecture to automatically create, update, and enhance static EA models with runtime information. These authors propose a new integration layer that synchronizes static and runtime data from different data sources. In the same line, Sánchez et al. [54] provided an technique to collect “information from multiple sources such as information systems, databases, files (system’s logs, source code, configuration), and previously existing models” and create enterprise models in a semi-automatic manner. Other proposals employ process mining techniques with runtime execution data to visualize the respective runtime enterprise architecture [63]. Similarly, Liu et al. [31] recover component-based architectures from software execution data. Truong et al. [62] proposed a method that combines “enterprise's strategy together with data mining rules extracted from the data warehouse of the enterprise in order to make design-time changes to its business processes”. Werf et al. [65] also consider operational data for extracting architectural descriptions in which quality attributes are considered apart from functional aspects. Johnson et al. [25] proposed to use dynamic Bayesian networks for automating EA modelling, which was then specifically realized for ArchiMate models by [5].

In contrast with our research, all the mentioned works do not follow a clear MDE approach. In this sense, there exist some works that generates EA models by following the MDE principles. For example, Ge et al. [17] attempt to model a system-of-systems (SoS) architecture framed in the DoDAF Metamodel. This work facilitates the automated transformation of executable models from architectural information. Also, Hu et al. [22] define an MDE method for service oriented SoS architecting, modelling and simulation. This work employs SysML to cope with the intrinsic complexity of SoS and make it possible the alignment of business requirement and IT infrastructure. Bogner and Zimmermann [6] use some metamodeling principles together with some ontology-based methods for the integration of microservices architectures. Similar to this work, Granchelli et al. [18] employ a domain-specific language (DSL) to automatically represent microservices architectures.

All the previous works (in the context of MDE) do not employ KDM as the core metamodel to manage a common knowledge repository. There are other works using KDM and model transformations in the context of MDE. For example, Landi et al. [30] define a DSL based in the *Structure* KDM layer for representing planned architectures, i.e., not only the architectural abstractions of the system but also the access rules that must exist between them and be maintained over time. Moutaouakkil and Mbarki [32] define a KDM extension to represent the Model/View/Controller (MVC) architectural concepts of web application into KDM models. However, this information is not used to generate EA models. Pérez-Castillo et al. [44] provide an ADM framework based on KDM to generate business process models from KDM models, which are previously obtained through various sources, for example, from source code [42, 50], data models [46], or events logs [50].

Finally, there are other works that provide model transformations between some EA concepts or artefacts and ArchiMate models. For example, Buckl et al. [8] propose an approach based on model transformations implemented in QVT to transform EA data to their graphical representation. Today, the creation of visual EA models is already solved through the majority of EA suites. Our current proposals focus on generating EA models from other artefacts by reverse engineering. Other work employing model transformations is proposed by [11]. However, those model transformations are actually metamodel mappings with fictitious model transformations, i.e., such transformations are not coded in a model transformations language like ATL or QVT as our proposal is. Thus, such transformations cannot be executed automatically. Engelsman et al. [13] propose some guidelines for transforming business models into EA models, with which to improve the traceability of the contribution of IT to the value offerings of a business. However, this transformation has not been implemented. Holm et al. [21] propose an approach and a tool to generate EA models (using Archimate as example) based on network scanning for recovering data automatically and then mapping this data to EA elements. This approach needs to manually define the mappings within the tool to generate the EA model each time (for each language used, i.e., Archimate) with no use of KDM, where the transformation is implemented in the tool. This makes the usability, extensibility, and changeability somehow limited. Opposite our approach provides clear rules for mappings defined in ATL which is easy to extend for new mappings, as presented in Sect. 4.3. Pepin et al. [41] a software modernization approach is taken to link legacy software architecture models with enterprise business models via KDM and using MoDisco [7] and ATL transformations to generate Application, Functional and Business Process models. However, the metamodels used are not standard (i.e., not Archimate or BPMN 2.0) making it difficult for organizations to integrate into their models. Differently, our approach is completely based on existing standards both for models and for transformations.

## Research proposal

The research method used is Design Science Research Method (DSRM) [20, 24, 40, 66]. DSRM proposes a set of steps or activities to complete the design and construction of some artefacts. The piece of research in this article (the KDM-to-ArchiMate transformation) is classified as “development- and evaluation-focused design science research”. This is owing to the fact this research is mainly concentrated in the three last activities of the DSRM (i.e., design and develop, demonstrate and evaluate). This DSRM scenario is aimed at designing and developing an artefact using both research and creative methods, as well as a demonstration and a thorough evaluation by means of experiments, case studies or other research strategies.

The artefact under investigation is a method for extracting EA models (represented using ArchiMate) from KDM models. In order to understand how KDM models are generated, Sect. 4.1 introduces the generic technique to obtain ArchiMate models from information systems’ artefacts. Then, the generation of KDM models specifically from source code is detailed in Sect. 4.2. Finally, Sect. 4.3 covers the main goal of this research, the model transformation between KDM and ArchiMate.

### General method for reversing ArchiMate models

**Fig. 5:**
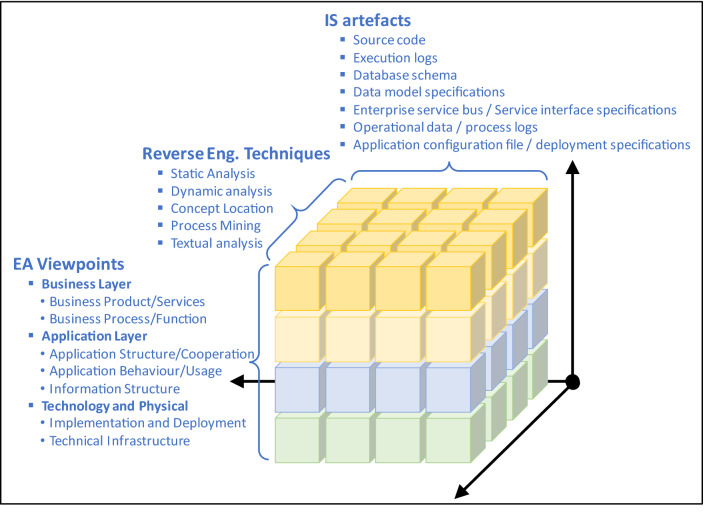
Overview of ArchiRev dimensions

ArchiRev is the method for extracting EA Models which has been proposed in a previous research [48]. ArchiRev considers ArchiMate for modelling EA. This method is generic and extensible since it is based on a set of reverse engineering techniques aimed at generating ArchiMate models by analysing software artefacts. In this method, different software artefacts can be considered as input by using specific and/or adapted reverse engineering techniques which, in turn, can discover and model further EA elements. Different reverse engineering within ArchiRev not only contribute to generate more accurate and complete EA models (i.e., further elements). Additionally, such techniques take into account certain information of IS artefacts to generate specific viewpoints concerning different stakeholders (see Fig. 3). It should be noticed that different EA views can be generated from EA models according to the viewpoints. Thereby, information gathered from IS artefacts drives the selection of certain elements to be included in a specific view, as well as some relationships between those elements. In these specific viewpoints, some layout issues could be addressed through reverse engineering techniques included in ArchiRev. Therefore, ArchiRev can be understand as a three-dimensional approach with three different dimensions that can be considered (see Fig. 5).

These dimensions are (i) the possible EA viewpoints/concerns that ArchiRev is able to extract and generate; (ii) the possible reverse engineering techniques that could be used to extract some of those specific EA concerns/viewpoints; and finally (iii) the dimension of IS artefacts that are considered during reverse engineering for gathering relevant EA information. Table 1 shows the most common possible combinations between systems artefacts (in rows), reverse engineering techniques (columns to the left) and the EA viewpoints according to Archimate (columns to the right).

**Table 1:** ArchiRev mapping regarding most common relevant use of reverse engineering techniques, input IS artefacts and EA viewpoints generated

Input IS artefacts	Reverse Eng. Techniques					EA viewpoints by layer						
	Static analysis	Dynamic analysis	Concept location	Process mining	Textual analysis	Business layer		Application layer			Technical layer	
						Business product/services	Business process/function	Application structure/cooperation	Application behaviour/usage	Information structure	Implementation and deployment	Technical infrastructure
Source code	■	■	■					●	●	●	●	
Execution logs	■	■		■			●	●	●	●	●	●
Database schema	■				■		●			●		
Data model specifications	■		■					●		●		
Enterprise service bus/Service interface specifications	■				■	●	●	●	●			
Operational data/process logs				■			●					
Application configuration file/deployment specifications	■				■			●			●	●

### Generation of knowledge discovery metamodel repository

ArchiRev employs KDM to represent all the information extracted and generated through the analysis of information systems’ artefacts. In this way, all the knowledge is abstracted in a technological independent way.

**Fig. 6:**
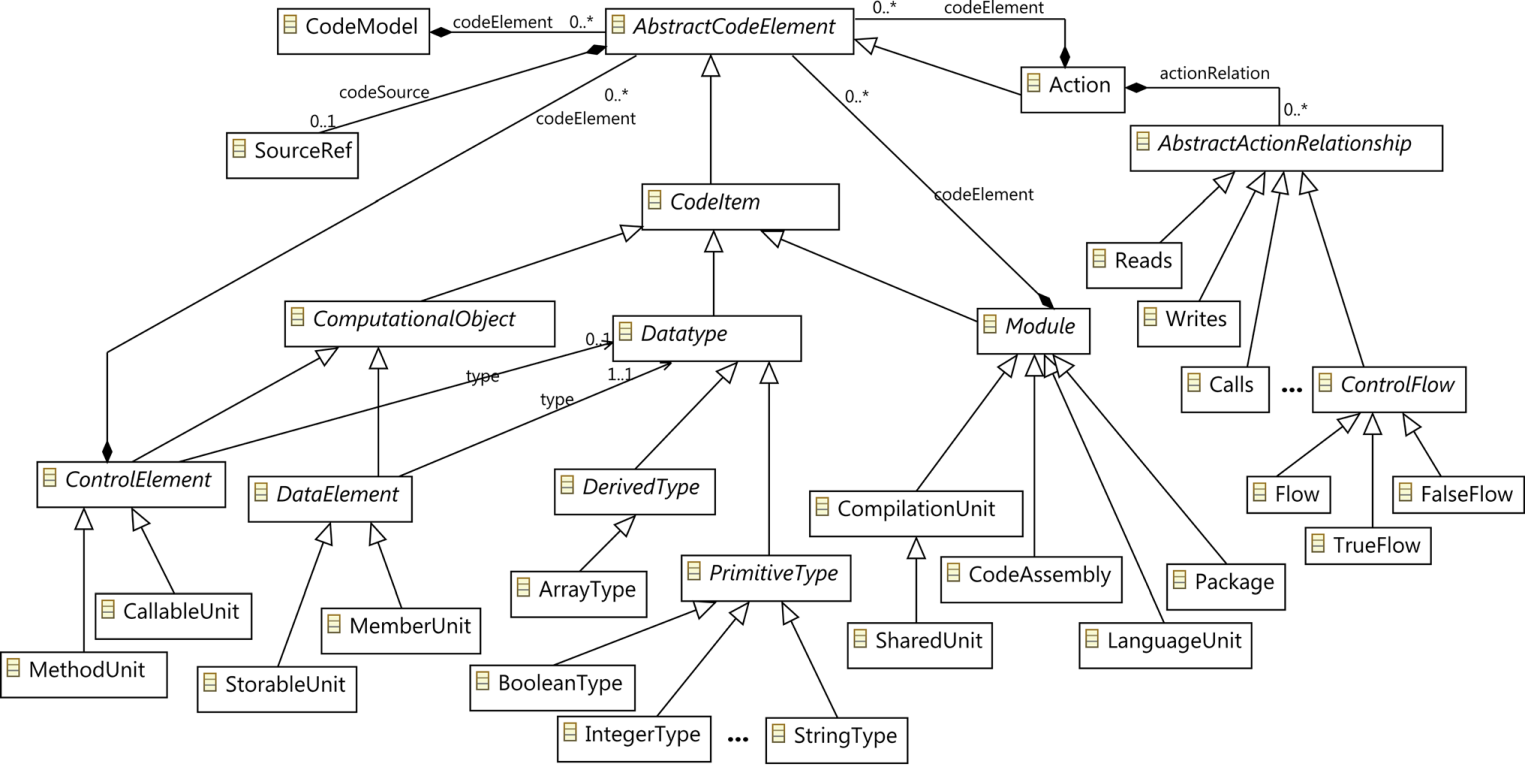
A simplified view of the Code and Action packages of the KDM metamodel

Different KDM packages and layers could be used depending on the artefacts analysed. The scope of the model transformation presented in this research is restricted to the *Code* and *Action* packages of the KDM metamodel, since we focus on source code. Further model transformations could be considered for other artefacts like data model, enterprise service bus, among other. The advantage of using KDM is that its metamodel covers the abstraction of different software artefacts. *Code* and *Action* within the *Program Elements* layer are the specific KDM packages to represent the source code are *Code* and *Action* (see Fig. 4). *Program Elements* is the second abstraction layer of KDM after the *Infrastructure* layer and it aims to provide a language-independent intermediate representation for various constructs determined by common programming languages. The *Code* package represents the named items from the source code and several structural relationships between them and the *Action* package focuses on behaviour descriptions and control- and data-flow relationships determined by them. Figure 6 shows the most important meta-elements of the *Code* and *Action* packages of the KDM metamodel. According to the KDM Code metamodel, each analysed source code artefact is represented as a *CodeModel* element, the root meta-element. A *CodeModel* is then composed as a set of code elements (*AbstractCodeElements*) such as *CallableUnit*, *StorableUnit,* and so on. The code elements can be interrelated among them (see AbstractActionRelationships) through relationships with different semantics such as *Flow*, *Calls*, *Reads*, *Writes*.

In addition to the elements shown in Fig. 6, other basic elements of the *kdm* and *core* packages in the KDM layers below are used in combination, for example, the *Annotation* element that allows textual descriptions to be attached to any instance of a model element. Information collected in these annotations that are attached to *CompilationUnit* elements are then a key source of information in the proposed model transformation.

For the case of source code, the most common technique employed to extract relevant information is static or dynamic analysis (see Table 1). Static analysis consists of a syntax inspection of the source code, which can be expressed in terms of a grammar. Commonly, we speak about parsers that are in charge of recognizing the whole structure of a piece of code and generate an abstract syntax tree (AST) from which specific information can be then gathered and represented in the KDM model (e.g., callable units belonging to a compilation unit). On the one hand, the advantage of static analysis is that there are many tools that support the automatic creation of specific parsers from grammars, which are available for the most common programming languages. On the other hand, static analysis fails to detect dead code (unreachable parts of source code) and to figure out parts of the code most frequently executed. Because of these inconveniences, dynamic analysis inspects the source code while it is being executed. Sometimes, source code is annotated with some statements able to register execution information, while other times profiling techniques (based on the execution environment) are used without altering the original artefact.

**Fig. 7:**
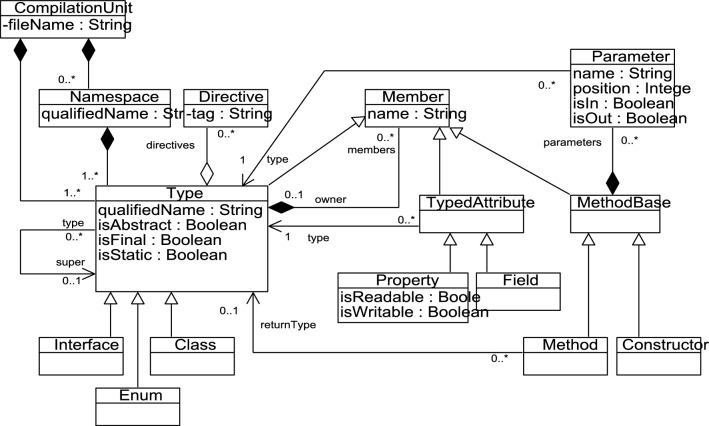
A simplified view of the C# metamodel

Within the context of ArchiRev, different parsers and dynamic analysers might be used in combination to inspect artefacts written in different programming languages. The derived information is then integrated according to the KDM metamodel. In the case study presented in the empirical validation (Sect. 5.2), we consider KDM models that are extracted from information systems written in C#. For this case, a parser has been coded based on the C# grammar. Specific implementation details are omitted in this paper since this kind of efforts has been extensively covered both in academia and industry. The recognized AST is built according to the C# metamodel depicted in Fig. 7. Then, a mapping is produced almost directly from the C# metamodel to the KDM metamodel previously depicted in Fig. 6.

### KDM to ArchiMate transformation

The M2M transformation from KDM to ArchiMate is based on one input and output metamodel. The input metamodel is the KDM metamodel defined in the standard ISO/IEC 19506 [45], while the output metamodel is the ArchiMate metamodel defined by Archi tool [4]. Although there is a tool-independent ArchiMate specification named Model Exchange File Format [61], we decided to use the Archi metamodel. The main drawback of not using the Model Exchange File Format is that it prevents the direct interoperability between EA tools. However, we believe that the usage of Archi metamodel has several advantages:

- The Archi metamodel provides a MOF-compliant metamodel, and Archi tool is based on ECORE metamodel. It offers an easier extensibility and integration with future model transformation through the Eclipse plug-in architecture and ECORE metamodels.
- Archi tool is an open-source tool used by a significant part of the EA community.
- Archi tool has been developed by The Open Group staff and is compliant with the ArchiMate standard.
- Archi can export ArchiMate models to Exchange File Format

The transformation from KDM to ArchiMate is based on the following mappings, which are shown in Table 2 for elements and in Table 3 for relationships between those elements. The whole model transformation implementation is available online [3]. The overall idea is that some of the compilation units in the KDM model are abstracted to a relevant software element in the target ArchiMate model. Also, associations and dependencies between these compilation units are analysed under some constraints to be filtered out and transformed into specific relationships between the respective ArchiMate elements.

**Table 2:** Mappings between KDM and ArchiMate elements

Compilation unit annotation	ArchiMate element
ManagedBean	Application function
Controller	Application function
Component	Application function
Named	Application function
Repository	Application component
SpringBootApplication	Application component
Service	Application service
Entity	Data object
Table	Data object
MappedSuperclass	Data object

**Table 3:** Default relationships between each pair of Archimate element kinds

Source	Target			
	Application Function	Application Component	Application Service	Data Object
Application function	Triggering	Triggering	Realization	Access
Application component	Serving	Serving	Realization	Access
Application service	Access	Access	Triggering	Access
Data object	Access	Association	Access	Composition

As it can be seen in Table 2, the mappings defined refer to a reduced set of elements from both metamodels, which are based on mappings provided in [43]. These mappings are based on the source code annotations and their semantics according to some software architectural patterns as well as according to some common coding platforms. However, mappings in Table 2 might be enhanced by adding more elements and the corresponding mappings to be used in the model transformation.

The mapping proposed considers four target ArchiMate elements (see Table 2): application functions, application services, application components and data objects. All these elements are in the *Application* layer of ArchiMate (see Fig. 3). This is due to the fact that the EA knowledge and semantics embedded in the source code (used as the input artefact) is mainly related to that layer. Application functions and components are generated from compilation units that, respectively, provides knowledge about the behaviour and the active structure. These elements represent the internal view of the EA model, while those related with the external view (such us application services and interfaces) are difficult to be generated from the source code information in the KDM model. Despite this fact, application services can be still generated from compilation units annotated as ‘services’. With regard to the collaborative behaviour (e.g., application interactions and application processes), these are not considered as target ArchiMate elements. Instead of this, the model transformation focuses on generating relationships between application components and applications functions. Finally, data objects represent the passive structure and can be mapped since the information of the usage of some data structures from the source code is available in the KDM model (e.g., some compilation units are representations of business/data entities in the source code).

The only origin element from the KDM metamodel that is taken into account is the *Compilation Unit* element, for which we analyse the type of annotation present in order to define to which element in the Archimate Metamodel it has to be transformed. When the annotation corresponds to *ManagedBean*, *Controller*, *Component*, *Named* and *Service*, the corresponding element is the *Application Function* element. When the annotation corresponds to *Entity*, *Table*, *MappedSuperclass* the corresponding element is the *Data Object* element, and when the annotation corresponds to the *Repository* or the *SpringBootApplication* element, the corresponding one is the *Application Component* element. For relations between elements, the corresponding type in Archimate for each one is shown in Table 3.

The transformation was implemented in ATL and takes as input a KDM model compliant with the KDM metamodel and generates as output an ArchiMate model compliant with the ArchiMate metamodel, which also includes the graphical representation of the elements. In the following, the transformation and its rules are described.

#### Transformation rules

The transformation defines mainly three types of rules, in which we separated three different creation types to organize the rules providing better understanding and extensibility:

1. From the *root* of KDM metamodel *Segment,* to the *root* of ArchiMate metamodel *ArchimateModel*, creating default *Folders* and *Viewpoints* to include graphical elements.
2. From *CompilationUnit* KDM elements to its corresponding ArchiMate elements, based on the annotation or name of the KDM element (cf. Table 2).
3. From relations between KDM elements into relations between ArchiMate elements, depending on the target elements that were generated (cf. Table 3).

#### Rule type 1: KDM segment to ArchiMate model

It generates the structure of the output ArchiMate model which will be populated with the corresponding elements depending on the input model. The structure of the output model will be the same for every input model, as a way to organize the resulting file. It will contain nine default folders which were selected based on the ArchiMate examples and most used categorizations for different elements: *Strategy*, *Business*, *Application*, *Technology & Physical*, *Motivation*, *Implementation & Migration*, *Other*, *Relations* and *Views*.

Although we generate the complete list of folders, since the mappings defined in Tables 2 and 3 refer to a reduced set of elements, at this point we only populate three folders:

- *Application* folder which contains the ArchiMate elements generated from the KDM *CompilationUnit* elements,
- *Relations* which include all the ArchiMate relations generated, and
- *Views* in which we generate the twenty-five *Viewpoints* proposed in the ArchiMate specification, where the graphical notation corresponding to the generated elements is included, for each element and relation between elements. Although only the *Application* and *Information Structure* viewpoints will be fully covered, the model transformation provides all the ArchiMate viewpoints as a predefined structure for helping with the future manual refinements by enterprise architects.

As mentioned before, each *ViewPoint* presents graphical diagrams containing selected types of elements, which can belong to several ViewPoints, and correspond to elements already generated in the model. As an example of the ViewPoints generated, we can mention: (i) *Information Structure*, which includes elements *Business object*, *Representation*, *Data object*, *Artifact and Meaning*, (ii) *Organization* which contains elements *Business actor*, *Business role*, *Business collaboration*, *Location* and *Business interface*. The complete list of ViewPoints and corresponding elements can be seen in the ArchiMate specification [59].

Listing 1 shows an excerpt of the *Segment2ArchimateModel* rule to present as example the generation of the root model and one view (*view1: Information Structure Viewpoint*) as well as one of the empty folder (*folder1: Strategy*) for the generation of the views and folders mentioned before.

#### Rules type 2: KDM CompilationUnit to Archimate elements

This type of rule deals with the generation of Archimate elements from KDM *CompilationUnit* elements, depending on the annotation or name it presents. To determine which rule will be applied we defined several helpers, which based on the KDM input element returns true on the type of annotation or name that it presents. Also, in these rules we added the generation of the corresponding graphical element that will be included in the associated viewpoint, under the corresponding View element that we created in the first rule.
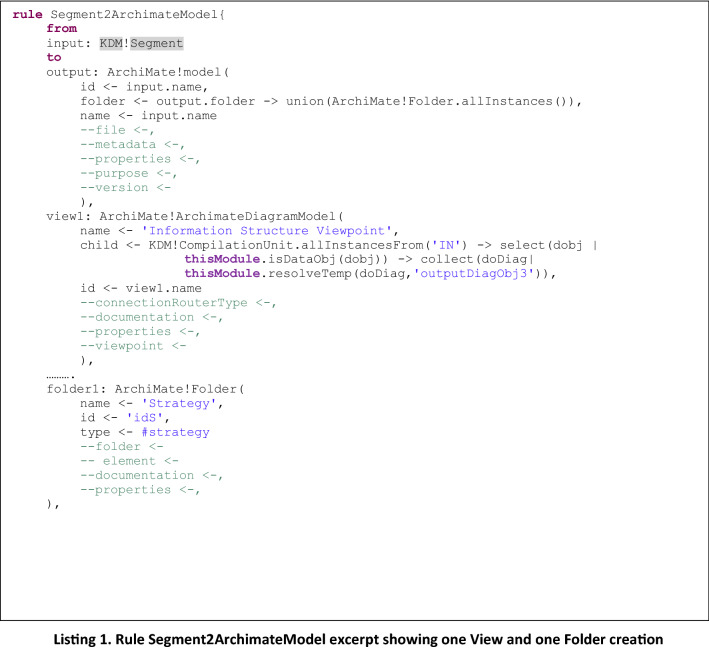


Each graphical element includes the Bound definitions (position and size inside the view), and the graphical representation of the relations for which the element is the source. Although the graphical representation of the relations is created in the type 3 relations rules, in the rules type 2 we are describing here, they are referenced in the corresponding element and connected to the general diagram. Anyway, visualization matters, like graphical and layout concerns, are outside of the scope of the model transformation. It simply takes default values since the visualization concerns is delegated in human modelers.

In Listing 2 we present an example of a type 2 rule, *CompilationUnit2ApplicationFunction* to generate Archimate *ApplicationFunction* elements from KDM *CompilationUnit* elements, and in Listing 3, the helper function that is invoked, which returns true or false depending on the type of KDM element that is being checked.

In Listing 4 another example of type 2 rule is presented, CompilationUnit2DataObject, to generate Archimate *DataObject* elements from KDM *CompilationUnit* elements, an in Listing 5, the helper function is presented, that is invoked from the rule and returns true or false depending on the type of KDM element that is being checked as input.
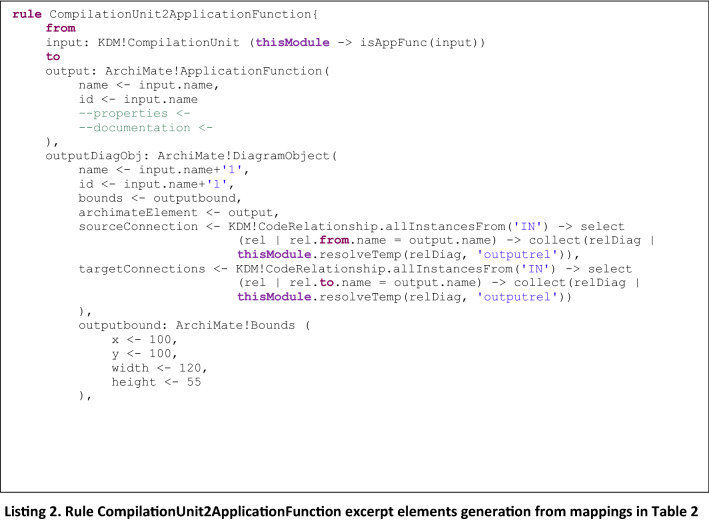



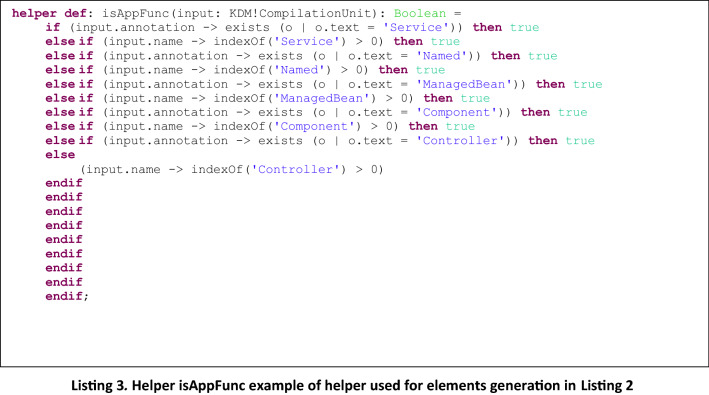



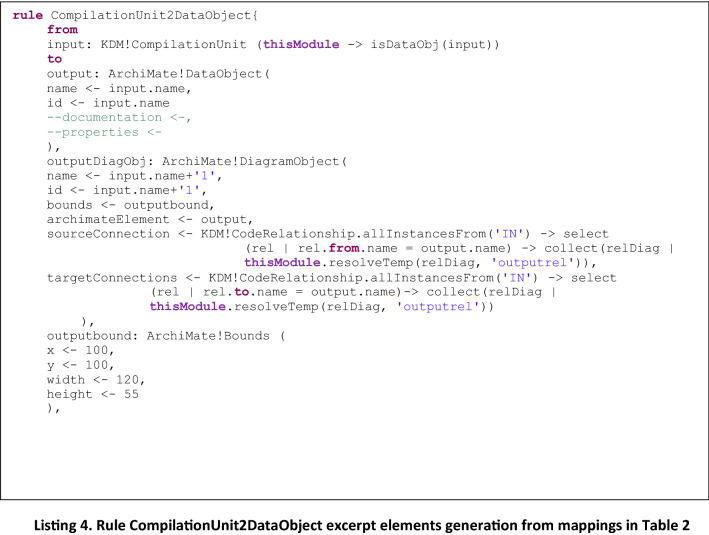



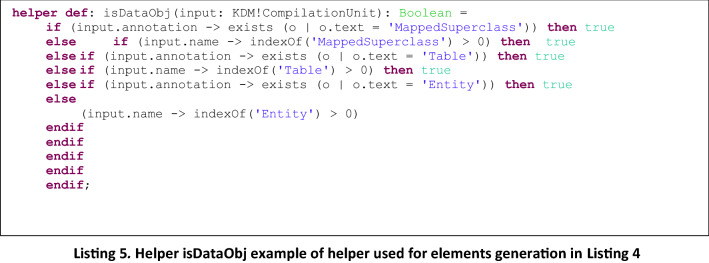


It is easy to note that adding a new rule to generate elements from a new mapping is straightforward: it only requires to add a new rule similar to the ones presented in Listing 2 and Listing 4, and define the corresponding helper to be invoked from the rule, which is in charge of checking whether the KDM input element corresponds to the desired one. So, the difference between the defined rules is provided by the helpers which allow the identification of specific elements from the KDM input Model, to be mapped to the corresponding Archimate output Model, as defined by the mappings presented in Table 2.

#### Rules type 3: KDM relations to Archimate relations

The type of the Archimate relation that is generated depends on the type of elements that are present in the source and target ends of the relation to use a relationship by default. The mapping that defines the type of relation is the one presented in Table 3, which is used in the rules to generate the corresponding type of relation, as well as the graphical representation of the relation. This is used as default relationship as the most common association between two kind of ArchiMate elements although this is not the only possible relationship that could be established according to ArchiMate. In these types of rules, we also defined helpers to check the type of relation that is under generation, and also include the graphical element and the reference to the corresponding existing Archimate element. In Listing 6 we present as an example a rule of a relation generation and in Listing 7 the corresponding helper.

#### Considerations

As mentioned before, the transformation is easily extensible to include new mappings for elements from KDM to Archimate, by adding the new rule only copying the structure of the type of rule that applies, and the corresponding helper to identify the input element and the corresponding output.


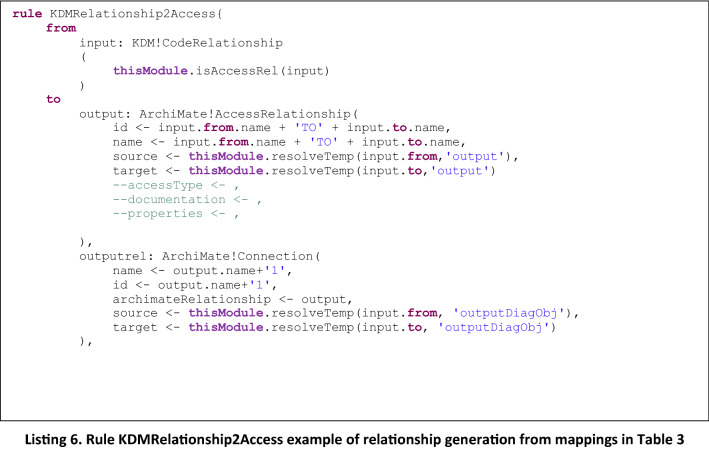



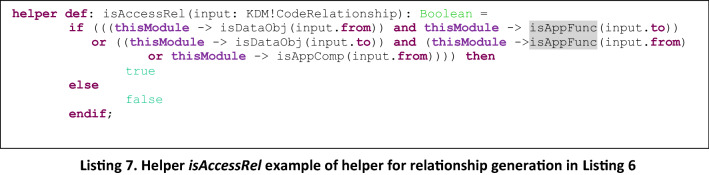


A compatibility problem with Archi tool was detected when generating the Archimate model. The *Ecore* metamodel used for the transformation, which we downloaded directly from the GitHub repository^1^ of Archi tool [4], was not exactly managed in the same way by Archi tool. The result was that some elements had different names in the *Ecore* metamodel and the model internally managed by the tool. This means that the output we generated (which we named with the *.archimate* extension to be imported by the tool) was not compliant with the tool metamodel and thus could not be opened in the tool.

To solve this, we decided to modify the Archimate metamodel we used in the transformation, by adding the elements needed for the tool to understand the output file. Specifically, we kept the base elements to preserve the compatibility with other Archimate files apart from those generated by our transformation. In order to use the original elements, we added:

- The *model* class as a copy of the *ArchimateModel* class
- The *DiagramObject* class as a copy of the *DiagramModelArchimateObject* class
- The *Connection* class as a copy of the *DiagramModelArchimateConnection* class
- The attribute *folder* inside the *FolderContainer* class as copy of *folders* attribute
- The attribute *element* inside the *Folder* class as copy of *elements* attribute
- The attribute *child* inside the *DiagramModelContainer* class as copy of *children* attribute
- The attribute *sourceConnection* and *targetConnection* inside the *Connectable* class as copies of *sourceConnections* and *targetConnections* attributes, respectively.

## Demonstration

To validate the automated generation of Archimate models from KDM models with our transformation, we used first a proof-of-concept to test and demonstrate the applicability of the model transformation (cf. Section 5.1), and then we conducted a more formal case study with six open-source information systems used for generating six KDM models used as input (cf. Section 5.2).

### Proof-of-concept

Before conducting the formal case study, we run a proof of concept with a real software application from which a KDM model was discovered through the ArchiRev Tool [1]. The use of this case study also allowed us to compare the generated model with previous generations that were carried out programmatically by means of a java tool that coded the transformation mappings in traditional structured programming. The result of that previous research is found in [43]. In order to facilitate the replication of this study, all the experimental materials (transformation, metamodels as well as input and output models) are available online [3].

The case selected was GIST-ERA, an information system of an Italian ship refurbishment company. GIST-ERA allows to manage and plan all the exact measurements that must be taken for every cabin. The particularity of huge cruise ships is that the size of cabin changes over time because of the metallic structure and continuous dilatation and contraction of this. GIST-ERA follows a client–server architecture. A client application for tablets allows the staff to register all the measures as well as help them to follow an optimal process preventing mistakes. The server side collects all the measures and manages them optimizing material and accessories orders. Technologically, the system is written in C# and uses a MS SQL Server as storage system. Additionally, this system was coded by using *Dynamic MVC* framework, which helps to produce data-driven Model/View/Controller applications. The size of the system is 38KLOC with 414 classes.

**Fig. 8:**
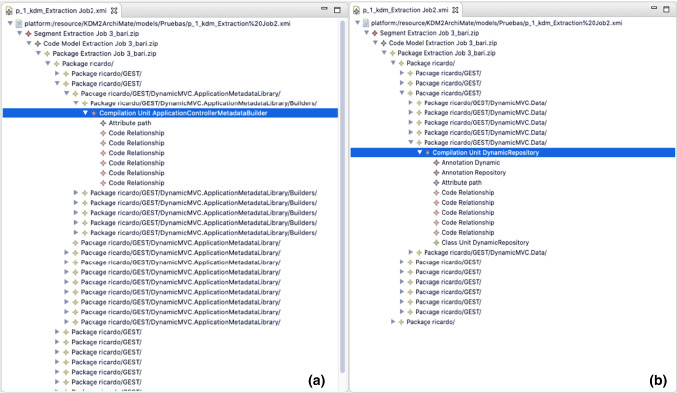
Input.xmi file for the KDM model with the ‘controller’ annotation in a CompilationUnit (**a**) and a ‘repository’ annotation in a CompilationUnit (**b**)

In Fig. 8 we present the input KDM model of the case study. In (a) a package where a *CompilationUnit* with the *Controller* word in the name is shown, which will be transformed into an *ApplicationFunction* in Archimate. In (b) a package where the *Repository* word is present as annotation and in the name of the element is shown, which will be transformed into a *DataObject* in Archimate.

**Fig. 9:**
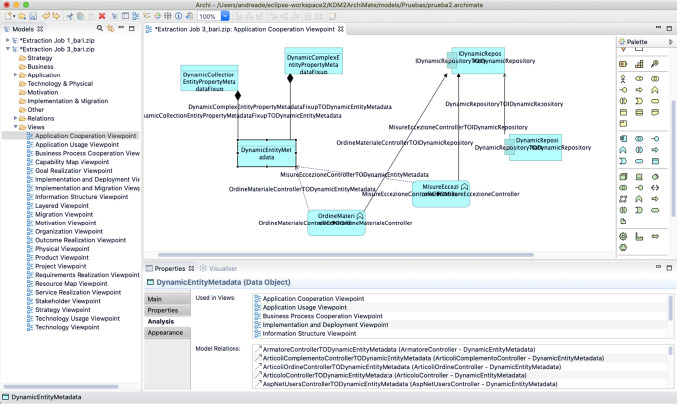
Excerpt of the Application Cooperation Viewpoint diagram of the generated ArchiMate model

After the execution of the transformation, we generated the ArchiMate model which is imported in the Archi tool and can be navigated within the generated folders, views, and other elements. Figure 9 shows a snapshot of the complete Archi tool with three main elements: the Models tree view can be seen on the left side, with the *Application Cooperation ViewPoint* diagram selected, showing the graphical representation of this viewpoint with an excerpt of the generated elements in the centre, and the elements palette on the right side of Fig. 9. The elements shown are of type *ApplicationComponent* in the top right (*IDynamicRepository* and *DynamicRepository*), *ApplicationFunction* in the bottom centre (*MisureEccezioneController* and *OrdineMaterialeController*), and *DataObject* in the top left (*DynamicComplexEntityPropertyMetadataFixup*, *DynamicCollectionEntityPropertyMetadataFixup* and *DynamicEntityMetadata*), with the last one selected. In the properties view it can be seen that the selected *DataObject* is used in many *Viewpoints* (*Application Cooperation*, *Application Usage*, *Business Process Cooperation*, *Implementation and Deployment*, and *Information Structure*) and presents several relationships with other elements, as shown in the Model Relations part.

### Case study

Although the proof-of-concept demonstrated the applicability of the model transformation to a real case, a more formal case study was conducted with six open-source systems.

#### Research goal and questions

The *subject* of the study is the ATL model transformation developed while the *purpose* of the study is the assessment of the complexity and the expressiveness of the outgoing ArchiMate models as well as the scalability of the model transformation, which are directly related to research questions RQ1 to RQ3, respectively.

- *RQ1*. Does the model transformation generate non-complex ArchiMate models?
- *RQ2*. Does the model transformation generate ArchiMate models with enough expressiveness?
- *RQ3*. Is the model transformation scalable for larger KDM models?

First, complexity (RQ1) has been widely studied as one of the most important measurable concepts in EA [23, 56], and it has been demonstrated to be related to the understandability and maintainability of models [9, 23, 53]. Second, expressiveness (RQ2) is introduced in this research as the ratio of input models that are eventually transformed into output elements. This aspect is important since the proposed model transformation filters some elements during transformation. Finally, scalability (RQ2) is related to the efficiency of the model transformation. This focuses on the scalability regarding the size of the input models to demonstrate its applicability even with larger systems. This is because we do not have benchmarks of similar model transformations to compare the transformation time.

It should be noticed that the effectiveness of the model transformation is not analysed from a point of view of the sensitivity and specificity of the ArchiMate elements generated (i.e., the study of recall and precision from an information retrieval perspective). This is because it is not possible to satisfy the need of human intervention, i.e., experts who know the context and business domain of every system and are able to annotate the ArchiMate models and determine wrong elements (false positives) and missing elements (false negative). This was done in a previous, similar case study [43]. However, to count with actual experts for open-source systems is no possible and this is the reason for which this is outside of the scope of the case study.

#### Measures and variables

The independent variable of the study is the case under study, i.e., the output ArchiMate model generated from each selected system, which is the unit of analysis. Regarding RQ1, the case study considers various variables that are directly related to the complexity:

- *Size* It is defined as the set of elements or relationships in the output ArchiMate model. It is distinguished for elements (Size_ele_) and relationships (Size_rel_). Size is an instrumental measure, but it is still associated with the complexity [23].
  $$\textit{Size}_{ele}=\#ApplicationFunction+\#ApplicationComponent+\#ApplicationService+\#DataObject$$
  $$\textit{Size}_{rel}=\#Triggering+\#Access+\#Association+\#Serving+\#Realization$$
- *Connectivity* It is the ratio between the total number of relationships and the total number of elements.
  $$\textit{Connectivity}=\frac{{Size}_{rel}}{{Size}_{ele}}$$
- *Density* It represents the ratio between the total number of relationship in a model and the maximum number of possible arcs (considering ArchiMate models as directed graphs). Both, connectivity and density affect the complexity (and therefore the understandability and maintainability) in a negative manner [9]. That means that lower connectivity and density values lead to ArchiMate models which are more understandable and modifiable, thanks to a lower level of intricacy.
  $$\textit{Density} = \frac{{Size}_{rel}}{\frac{{Size}_{ele}\cdot ({Size}_{ele}-1)}{2}}$$
- *Heterogeneity* (*Entropy*) This is applied to elements and relationships in ArchiMate models and measures the diversity of kind of elements or relationships used in a certain model. Heterogeneity is directly related to complexity [23, 56].
  $$\textit{Heterogeneity}_{ele}= -\sum_{e=1}^{n}{p}_{e}\cdot \mathrm{ln}\left({p}_{e}\right), { p}_{e}=\textit{relative frequency of element}\, e$$
  $$\textit{Heterogeneity}_{rel}= -\sum_{r=1}^{n}{p}_{r}\cdot \mathrm{ln}\left({p}_{r}\right), {p}_{r}=\textit{relative frequency of element}\, r$$
  With regard to RQ2, it evaluates the expressiveness that relates the output and input model. For this purpose, the measurable concept is the amount of class elements in the input model that are eventually transformed into one of the possible elements in the ArchiMate model. This attempts to provide a numeric value of the number of elements in the input models that were useful and therefore transformed into some elements in the output model.
- *Transformation ratio* It is defined as the ratio between the output size and the input size. The input size is defined as the number of *Class Unit* elements in the input KDM model.
  $$\textit{Transformation ratio}= \frac{{Size}_{ele}}{{Size}_{rel}}, {Size}_{input}=\#ClassUnit$$
  Finally, in order to assess RQ3, related to the study of the scalability, it considers the model transformation time to be analysed in comparison with Size_input_.
- *Model transformation time*, that is the total time spent by the ATL engine to execute the proposed model transformation and generate the ArchiMate model.

#### Case selection

The six case under study were selected according to the following criteria: (i) the system must be an enterprise system (i.e., the system supports business processes or some managerial aspects of the organization that uses the information system); (ii) it must be coded in C#, since this is the programming language supported by the ArchiRev tool to generate the KDM models; and (iii) the system must contain at least 5000 lines of code in C# to be analysed. Table 4 shows the six projects selected from GitHub with a brief description, KLOC, C# KLOC and the number of C# files to be analysed.

**Table 4:** Selected cases under study

ID	GitHub Project	Description	KLOC	C# KLOC	C# files
S1	go2ismail/Asp.Net-Core-Inventory-Order-Management-System	It is an inventory order management system. Warehouse, product, vendor, customer, purchase order, sales order, etc	1571	10	162
S2	SOFTENG701G1/Flatmate-Management-System	It manages flat (shared house) expenses for a given flat and tracking who has paid those bills	28	3	54
S3	nbarnwell/OrderManagementSystem	A sample application for manage orders	84	5	149
S4	cocoa-mhlw/cocoa	A COVID-19 Contact-Confirming Application (COCOA)	53	37	371
S5	trevoirwilliams/leave-management	Simple application for managing employee leaves	50	7	75
S6	M-Zuber/MyHome	A simple desktop program to manage home finances	20	13	122

#### Execution procedure and data collection

The execution procedure of the case study consists of four steps. First, (i) the source code of the information systems is analysed with ArchiRev and the respective KDM models are generated. Second, (ii) the KDM models are then transformed into ArchiMate models by means of the proposed model transformation that is executed through ATL engine embedded in Eclipse. Third, (iii) the ArchiMate models are inspected to take other measures that were not automatically collected (as the transformation time) and derived variables are computed as well. Finally, (iv) the whole dataset is analysed for answering research questions and draw conclusions of the case study.

The execution environment consisted of macOS BigSur with intel i5, ATL version 4.2.1.v202006221222 and Eclipse Modeling Tools version 2020-09 (4.17.0).

Table 5 shows the whole dataset completed after the six information systems (S1–S6) were analysed and transformed into ArchiMate models. First rows provide information about the input KDM models that was generated from the inspection of the C# source code. Then, the model transformation time in seconds is provides. The following set of rows provides number of elements and relationships in the outgoing ArchiMate model. Finally, bottom rows provide the three variables to be analysed: cohesion, coupling and coverage. The ride side of Table 5 provides aggregated values for every row with minimum, maximum, mean, and standard deviation.

**Table 5:** Dataset collected for the case study

	S1	S2	S3	S4	S5	S6	min	max	Mean	SD
*Input KDM elements*										
Package	293	17	73	115	22	37	17	293	92.8	104.7
Compilation unit	162	54	149	371	75	122	54	371	155.5	113.5
Class unit	191	44	143	327	94	86	44	327	147.5	101.5
Annotation	447	102	388	1057	288	202	102	1057	414.0	338.6
Code relationship	506	211	365	809	139	441	139	809	411.8	238.5
Transformation time (s)	1.634	0.704	0.301	2.024	0.100	0.412	0.10	2.02	0.86	0.78
*Output ArchiMate elements*										
Application function	65	11	20	82	4	16	4	82	33.0	32.3
Application Component	0	7	5	15	8	11	0	15	7.7	5.1
Data object	0	1	0	0	4	0	0	4	0.8	1.6
Triggering	66	31	20	144	5	53	5	144	53.2	49.7
Access	0	1	0	0	0	0	0	1	0.2	0.4
Association	0	1	0	0	0	0	0	1	0.2	0.4
Serving	0	13	8	24	14	21	0	24	13.3	8.7
*Metrics*										
Size_ele_	65	19	25	97	16	27	16	97	41.5	32.5
Size_rel_	66	46	28	168	19	74	19	168	66.8	53.9
Connectivity	0.98	0.41	0.89	0.58	0.84	0.36	0.36	0.98	0.68	0.26
Density	0.03	0.27	0.09	0.04	0.16	0.21	0.03	0.27	0.13	0.10
Heterogeneity_ele_	0.00	0.84	0.50	0.43	1.04	0.68	0.00	1.04	0.58	0.36
Heterogeneity_rel_	0.00	0.79	0.60	0.41	0.58	0.60	0.00	0.79	0.50	0.27
Transformation ratio	34%	43%	17%	30%	17%	31%	17%	43%	28.8%	10.1%

## Evaluation

This section provides the evaluation of the artefact proposed. First, Sect. 6.1 analyses the results obtained in the case study. Then, Sect. 6.2 discusses the threats to the validity.

### Result analysis

**Fig. 10:**
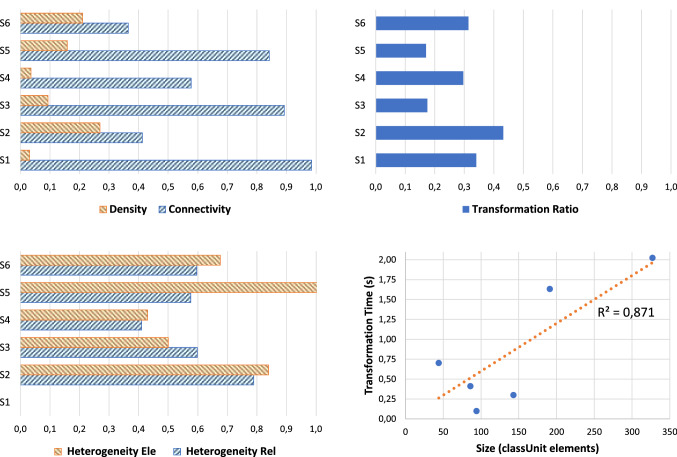
Summary of the analysis results for the case study

Figure 10 summarizes results of the case study. Left-hand side provides two bar plots for analysing variables regarding RQ1, i.e., connectivity and density (top-left) and heterogeneity (bottom-left). Then, the transformation ratio (RQ2) is graphically analysed in a bar plot (see top-right plot in Fig. 10). Finally, Fig. 10 (bottom-right) provides the scatter plot and trend line to analyse the scalability of the model transformation (RQ3).

Density is normalized between 0 and 1; and the values for the six models are 0.13 on average. According to the connectivity definition, these values are not normalized and could be higher for bigger models. In the 6 models with a Size_ele_ = 41.5 on average, the connectivity values vary between 0.36 and 0.98. Although there are not indicators in the literature for connectivity, the obtained values mean that there are between one and three elements for each relationship in the ArchiMate Model. These results suggest that the complexity of the model is affordable.

With regard to heterogeneity, the six models have similar values on average (see Fig. 10) that are, respectively, 0.58 and 0.50 for elements and relationships. On the one hand, these values are low contributing to a lower complexity which is good, in turn, for understandability and maintainability of ArchiMate models. On the other hand, it should be noticed that there are various kind of ArchiMate elements that are almost not present in any of the six output models (see Table 5). This suggests that additional mappings considering other input annotations (apart from those in Table 2) could be necessary to be able to generate more elements and provide more complete ArchiMate models. Involving human experts in future case studies will probably lead to improve the model transformation in this regard. However, as we explained before, it is outside of the scope of this current study.

As a result, RQ1 can be answered positively, although in a moderate way. The results obtained seems to provide ArchiMate models with a moderate complexity that in somehow are manageable by enterprise architects in case these models have to be improved, modified or integrated with other EA models.

About RQ2, the transformation ratio in some of the output models is medium–low, with an approximate 30% on average. This signifies, that various class units in KDM were not used in the model transformation rules. This was expected since class units are filtered according to specific annotations as we previously depicted. In other words, a transformation ratio of 100% was not expected. Thereby, this value is certainly representative. In general, we cannot reject the hypothesis that the model transformation does not provide output model with enough expressiveness. Anyway, further experimentation will be necessary as we already mentioned.

About scalability (RQ3), despite we have few cases to extract stronger conclusions, the trend line according to the correlation value (*R*^2^ = 0.87) suggests a linear relationship between the size of KDM models and the time spent to transform such models. As we discussed before, the model transformation could vary depending on the transformation ratio (i.e., the number of annotation in class units more than merely depending on the number of class units) among other factors. Anyway, with the current evidence, we can suggest that the model transformation time will not increase exponentially for larger KDM models. Actually, the *R*^2^ for the exponential model was 0.41, which can explain worse the hypothesized scalability.

### Threats to the validity

The case study has some issues threatening its validity that must be commented transparently. First, the case study considers variables that have been used in similar works directly related to the complexity of EA models; however other measures based on the experts’ opinion could improve the evidence about effectiveness of model transformation (e.g., precision and recall regarding relevant and missing elements in output models among others). Although it is a threat for the construct validity, the lack of available experts on eligible cases prevents to choose these metrics.

Regarding internal validity, there is no large population with regard to the cases under study. Therefore, results are statistically less representative. Despite this, a trend for the proposed measures was identifiable in the case study. In order to mitigate this threat, we hope to contrast the result of this case, by means of meta-analysis, with future results obtained from additional case studies.

Other threat to the internal validity is the tool we employed for generating the KDM models (ArchiRev), since it introduced a bias for the study. KDM models representing KDM *code* and *action* packages that are generated with other tools might be included in future case studies. Also, the outgoing EA models are not represented with the Model Exchange File Format which prevents the tool interoperability and therefore limits the generalisability of results.

Finally, about generalisability of the results, it can be only generalised to KDM models generated with ArchiRev from enterprise/management information systems coded in C#. Thus, it is clear that further evidence is necessary.

## Conclusions and future work

One of the biggest challenges to achieve an operational Enterprise Architecture Management is the ability to automatically retrieve parts or skeletons of enterprise architecture models from the most common IT artefacts. We believe the semiautomatic EA modelling is key to re-align business and IT in a volatile business world. We think an MDE approach helps a lot in this matter. Thus, the research presented in paper follows the MDE approach and proposes the usage of KDM as an intermediate step between information systems artefacts and target ArchiMate models. The usage of KDM helps to integrate knowledge extracted by reverse engineering from different artefacts. As a result, the usage of KDM contributes to integrate information coming from different sources and thus the proposed model transformation between KDM and ArchiMate can exploit those cross-cutting relationships.

The KDM-to-ArchiMate model transformation has been implemented in ATL, which allowed us to validate it with a KDM model extracted from real-life information systems. We believe this case study demonstrates the feasibility of transforming KDM into ArchiMate models which, in turn, facilitates the applicability in the industry in a greater extent.

Despite the preliminary insights, we are conscious of the limitations of the proposal. For example, KDM should be populated with the extraction of information of further information systems’ artefacts like for example, data model, enterprise service bus, etc.; and the ATL model transformation should be extended accordingly. Fortunately, KDM is an ISO/IEC standard that is employed in the industry up to a certain extent, so many reverse engineering tools that are able to generate KDM models may be reused, i.e., the outgoing KDM models may be transformed into ArchiMate models. As a consequence, EA mining is automated, and the EA management is benefited through an easier, continuous re-adaptation. In this way, the EA debt [19], analogous to the technical debt, can be kept or even reduced. Also, one of the rationale of this proposal was that the usage of KDM prevent to use independent silo solutions for EA modelling. Thus, a limitation of this work is the lack of validation to demonstrate that a KDM-based approach performs better than silo solutions. This means that the usage of MDE and standard metamodels on the automatic EA modelling should be analysed in the future to figure out how those aspects influence the mentioned problems of manual EA modelling, i.e., error-proneness, time-consumption, slow and poor re-adaptation and costs.

Our future research will be basically oriented toward improvement of the model transformation through the parametrization. We are conscious of default relationships defined between certain types of ArchiMate elements could be improved with some previous setup that allow to generate different types of relationships under different conditions. Moreover, we will work on strengthening the validation of the model transformation with additional case studies in different information systems and using further IT artefacts.

## Footnotes

1 ArchiMate Ecore: https://github.com/archimatetool/archi/tree/master/com.archimatetool.model/model.
